# Supplementary material for: PD-L1 Testing and Squamous Cell Carcinoma of the Head and Neck: A Multicenter Study on the Diagnostic Reproducibility of Different Protocols
Source: Cancers (Basel). 2021 Jan 14;13(2):292. doi: 10.3390/cancers13020292 (PMC7830149; doi:10.3390/cancers13020292)
Supplement: Supplementary file 1 [file cancers-13-00292-s001.pdf]

cancers

Supplementary Materials: PD-L1 Testing and Squamous Cell Carcinoma of the Head and Neck: A Multicenter Study on the Diagnostic Reproducibility of Different Protocols

Simona Crosta, Renzo Boldorini, Francesca Bono, Virginia Brambilla, Emanuele Dainese, Nicola Fusco, Andrea Gianatti, Vincenzo L'Imperio, Patrizia Morbini and Fabio Pagni

Table S1. CPS extended results. ne, not evaluable.

N	Protocol 1	Protocol 2*	Protocol 3	Protocol 4	Protocol 5	GS
1	90	>20	80	100	100	100
2	ne	ne	ne	ne	ne	ne
3	0	1–20	0	1	0	3
4	0	<1	0	0	ne	0
5	15	>20	35	82	40	19
6	ne	ne	ne	ne	ne	0
7	ne	<1	ne	ne	0	0
8	60	>20	10	26	50	40
9	50	>20	30	14	60	25
10	80	1–20	15	4	5	25
11	ne	ne	ne	ne	ne	0
12	80	>20	70	100	100	90
13	5	1–20	1,5	2,5	0	1–20
14	10	1–20	8,5	10	5	>20
15	15	>20	12,5	12,5	15	>20
16	10	>20	11,5	12,5	25	15
17	0	< 1	0	0	0	0
18	0	< 1	0	1	0	0
19	ne	ne	ne	ne	ne	ne
20	0	<1	5	8	0	10
21	ne	<1	ne	ne	ne	0
22	70	>20	80	100	100	100
23	2	1–20	0	0	0	8
24	10	<1	70	100	95	0
25	85	>20	80	100	100	>20
26	ne	ne	ne	ne	ne	>20
27	ne	ne	ne	ne	ne	ne
28	60	>20	45	60	75	>20
29	5	1–20	4	20	3	15
30	10	1–20	3	5	1	5
31	ne	ne	ne	ne	0	1–20
32	18	>20	30	30	100	>20
33	5	>20	4	60	20	>20
34	0	1–20	0	6	7	>20
35	2	<1	2	12	3	15
36	5	1–20	9	13	20	>20
37	0	ne	0	0	0	0
38	0	ne	0	15	0	0
39	0	<1	3	30	1	ne
40	15	1–20	0	2	0	10

* Results were expressed in reference ranges due to the peculiar characteristics of SP142 staining.

© 2021 by the authors. Licensee MDPI, Basel, Switzerland. This article is an open access article distributed under the terms and conditions of the Creative Commons Attribution (CC BY) license (http://creativecommons.org/licenses/by/4.0/).
